# Supplementary material for: Pink-beam serial crystallography
Source: Nat Commun. 2017 Nov 3;8:1281. doi: 10.1038/s41467-017-01417-3 (PMC5668288; doi:10.1038/s41467-017-01417-3)
Supplement: Supplementary file 1 — Supplementary Information [file 41467_2017_1417_MOESM1_ESM.pdf]

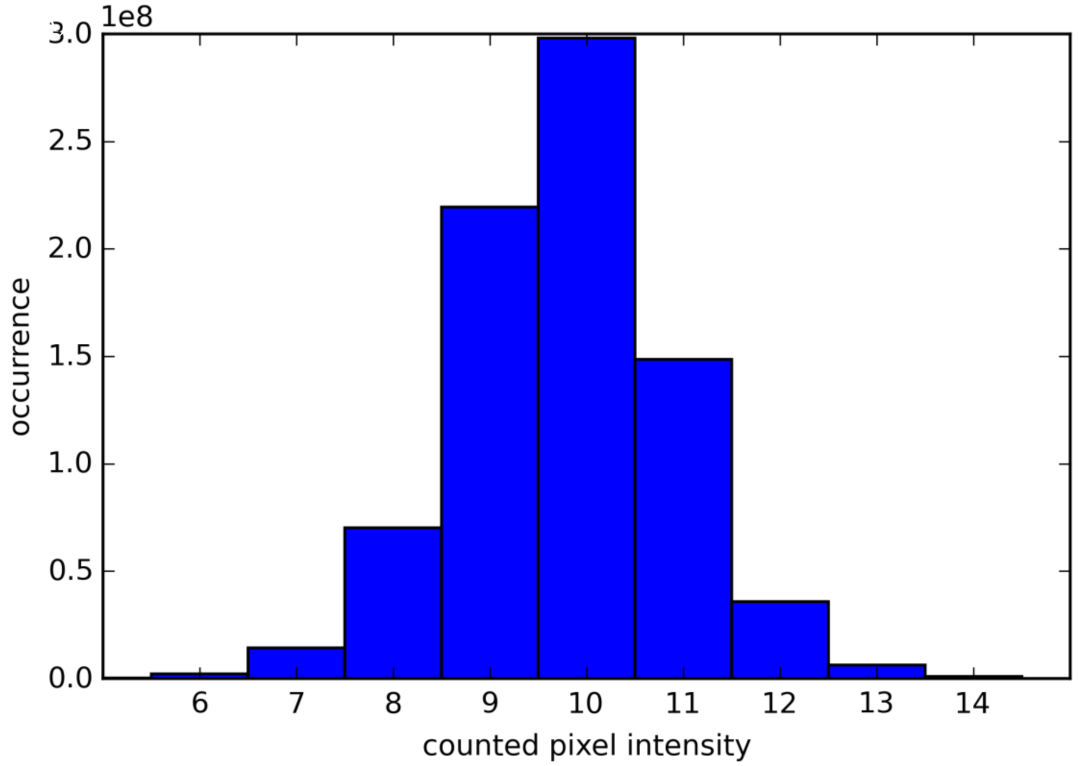

Supplementary Figure 1: Histogram plot showing the read-out noise statistic of the Rayonix MX340HS CCD from a dark image. The standard deviation of 2.43 ADU's is five times higher than the scattering background from our setup.

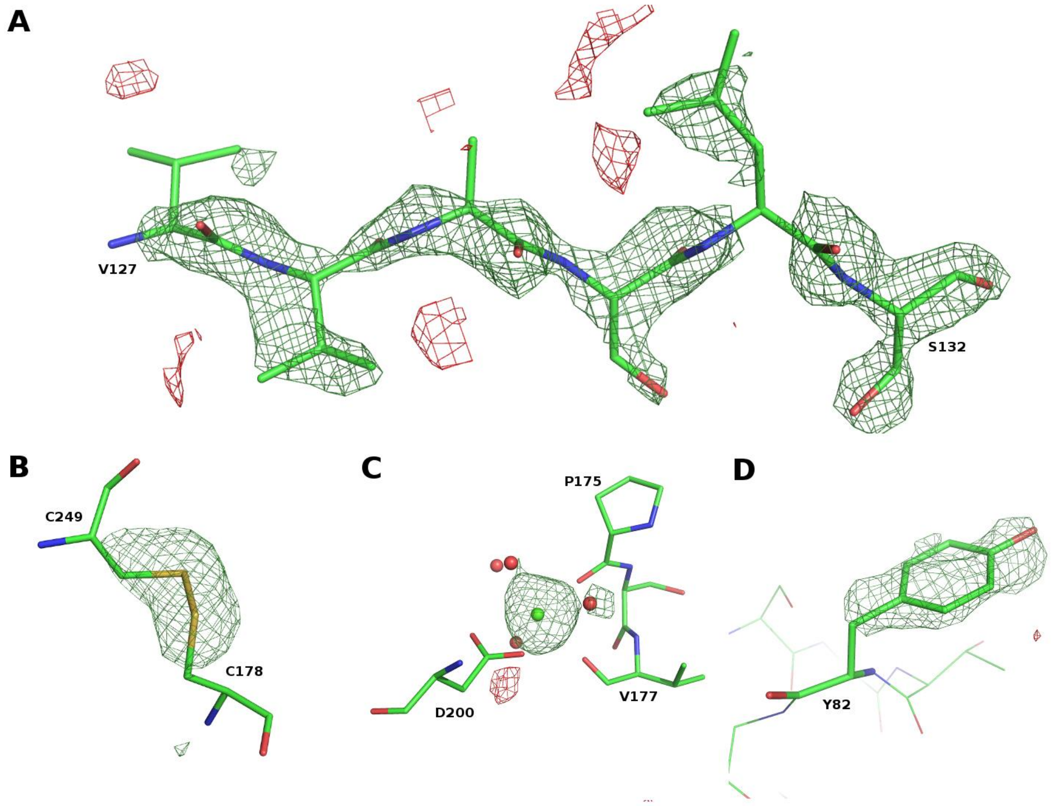

Supplementary Figure 2: Simulated annealing omit maps of proteinase K. To exclude potential model bias, electron density simulated annealing omit maps were used during refinement of the proteinase K structure derived from single shot pink beam serial crystallography experiments. The exemplary mF_o-DF_c simulated annealing omit maps represented here as green/red grid at a contour level of 3σ, were obtained by modification of the final model of the proteinase K structure followed by simulated annealing refinement and map calculation with Phenix.refine. The atom occupancies of certain amino acid residues were either set to zero (A: residues V127 to S132A; C: Ca^{2+} ion and surrounding water molecules) or the residues were mutated (B: C249A, C178A; D: Y82A). The positive peaks of the omit maps are in good agreement with the final structural model of proteinase K (green sticks). The disulfide bridge (A) shows no sign of radiation damage.

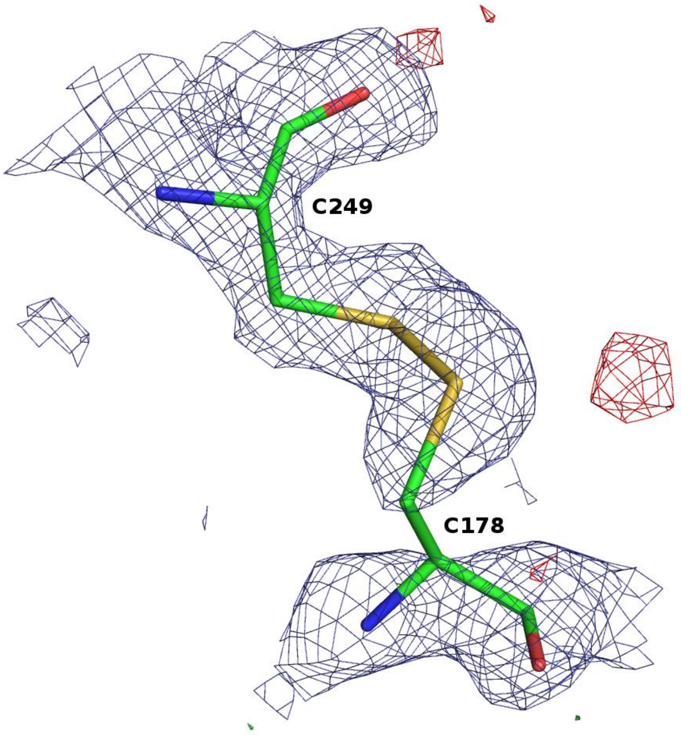

Supplementary Figure 3: Exemplary electron density map of a disulfide bridge of the proteinase K structure derived from single shot pink beam serial crystallography experiments. The $2mF_o$-DF_c map at a contour level of 1.5σ is shown as blue grid, the mF_o-DF_c map at a contour level of 3σ in green/red. The absence of negative mF_o-DF_c density around the sulfur atoms of the disulfide bridge indicates that there is no specific radiation damage in the proteinase K structure.

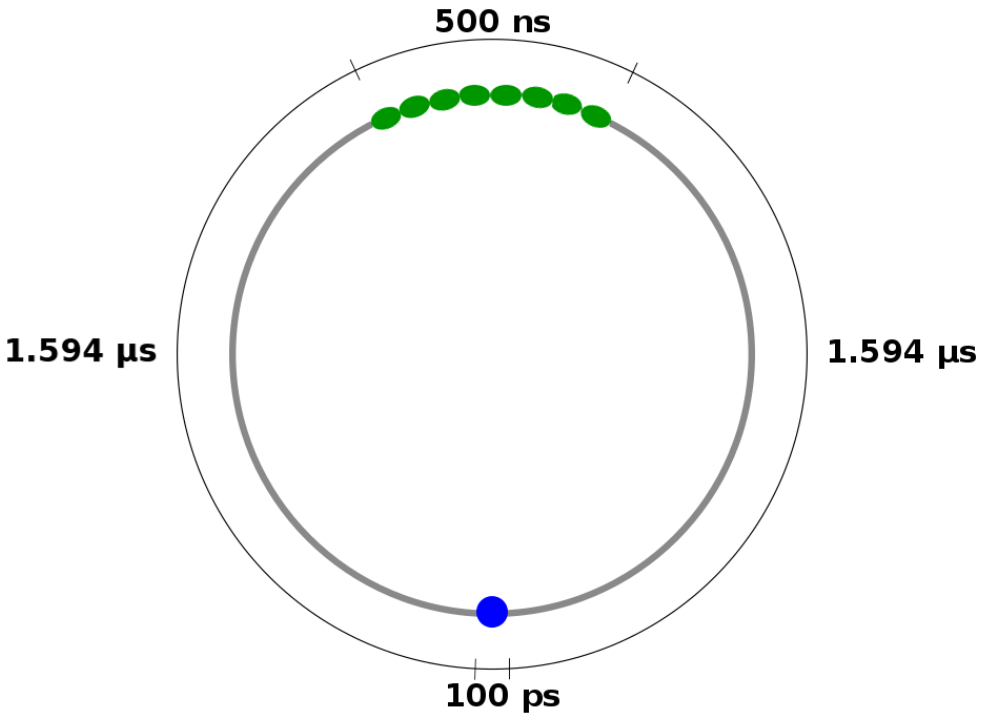

Supplementary Figure 4: Schematic of the hybrid filling mode at APS. A single electron bunch with 16 mA charge and a duration of ~100 ps (blue) is separated from a bunch train consisting of eight septuplets with a total charge of 86 mA and a duration of 500 ns (green). The separation between the single bunch and the nearest septuplet is 1.594 µs in either direction. Adapted from ref. [1]

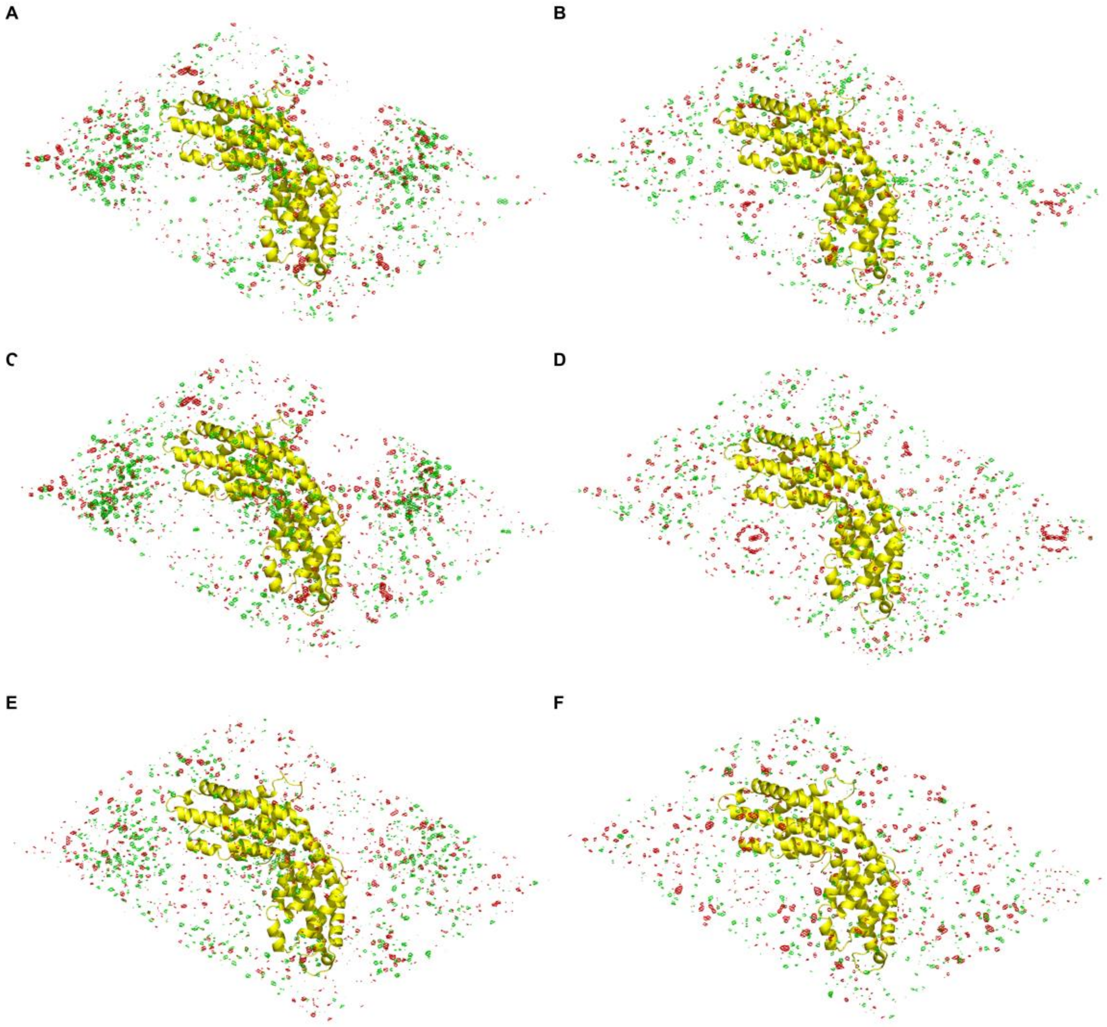

Supplementary Figure 5: Exemplary F_o-F_o-maps between different phycocyanin datasets: A) $F_{O,Phyco\_A1}$– $F_{O,1JBO}$, B) $F_{O,Phyco\_A1}$ – $F_{O,4ZIZ}$, C) $F_{O,Phyco\_B}$ – $F_{O,1JBO}$, D) $F_{O,Phyco\_B}$ – $F_{O,4ZIZ}$, E) $F_{O,Phyco\_C}$ – $F_{O,1JBO}$ and F) $F_{O,Phyco\_C}$ – $F_{O,4ZIZ}$. All maps are displayed for one unit cell, in the standard view when starting PyMOL from Phenix. All maps are contoured at 3σ and for all maps the structural model from PDB-entry 1JBO was used for phasing. Corresponding CC_{iso} and R_{iso} values can be found in supplementary Table 1. No residual F_o-F_o difference electron density can be found around the Sulphur atoms in phycocyanin and most difference density peaks are – as expected - in proximity to long flexible amino acid side chains.

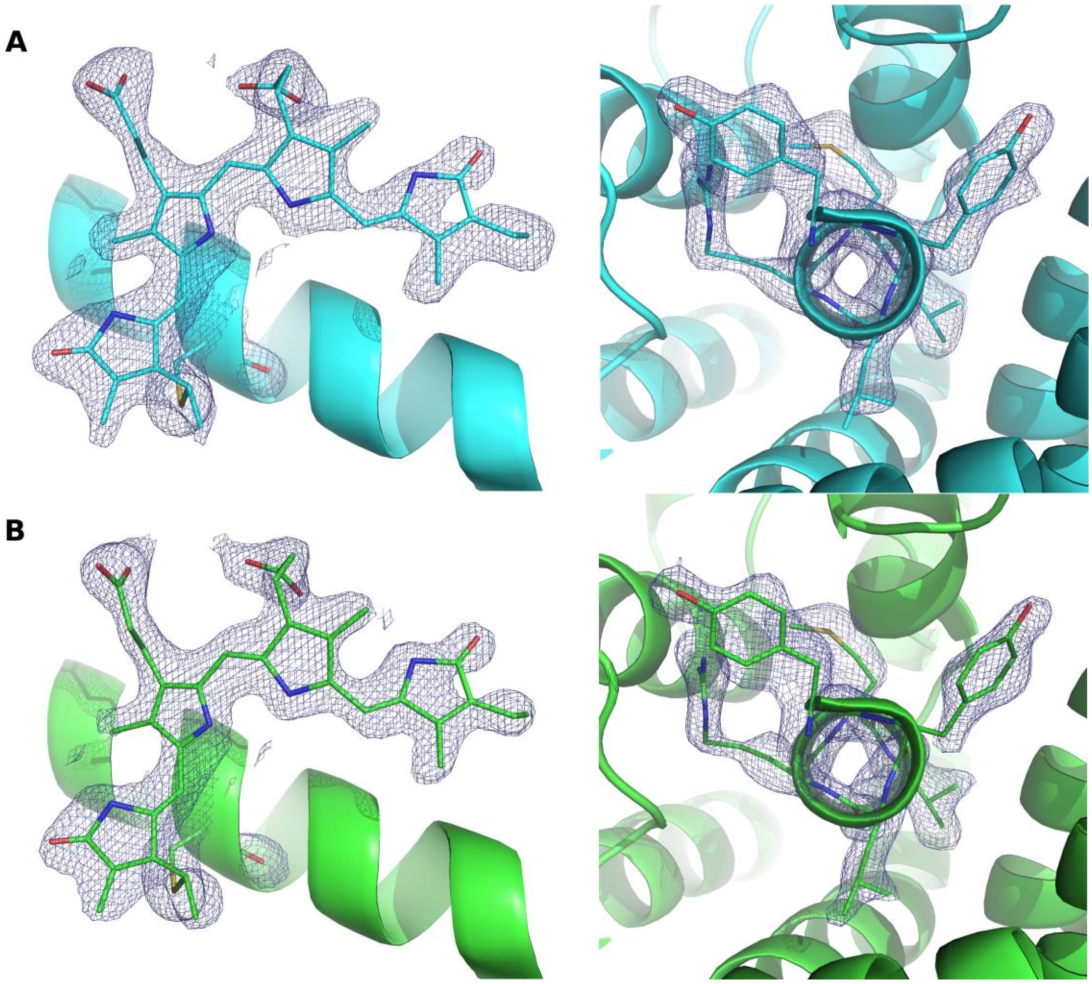

Supplementary Figure 6: Exemplary electron density of phycocyanin structures obtained from pink beam serial crystallography experiments. The blue grids represent $2mF_o$-DF_c simulated annealing composite omit maps at a contour level of 1.5σ. One phycocyanobillin molecule covalently bound to α-helix 6 is shown on the left and a view along the axis of α-helix 6 can be seen on the right. (A) Electron density from dataset Phyco_B obtained from the measurement of 205 diffraction images using the APS single bunch. (B) Electron density obtained from 52 diffraction images using a longer exposure time of 3.68 µs per crystal (Phyco_C).

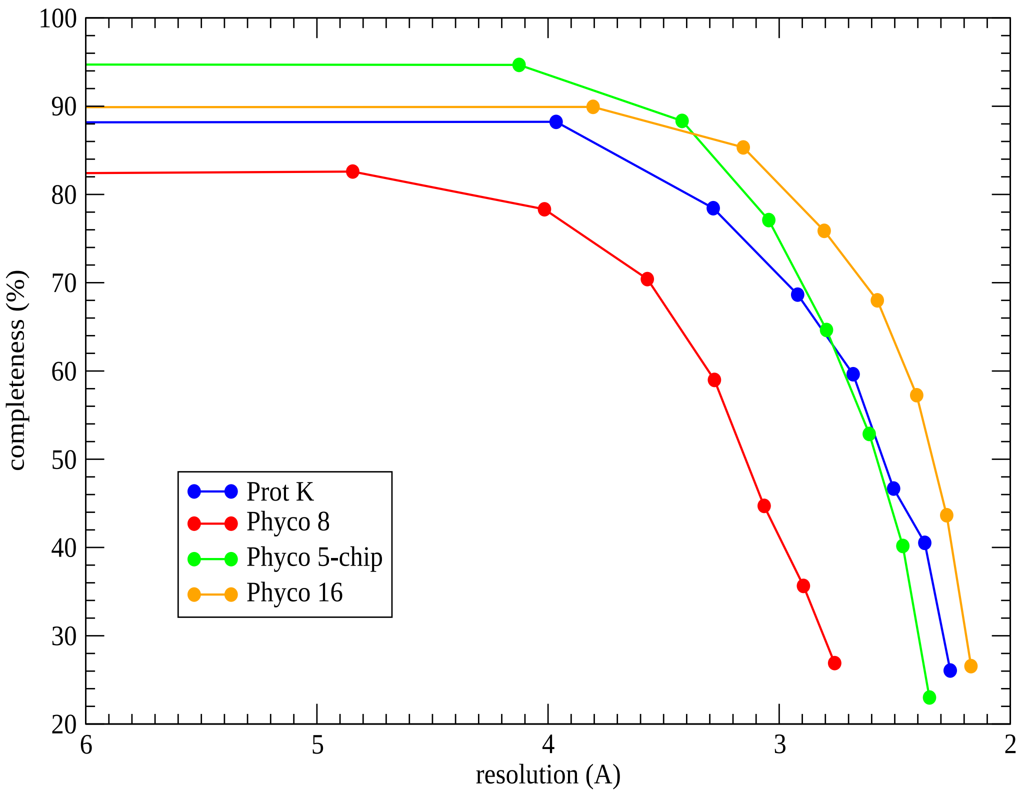

Supplementary Figure 7: Completeness of proteinase K and the three different phycocyanin datasets from Table 1 as function of resolution. Only reflections with an I/σ(I) ratio larger than 3 were considered.

A

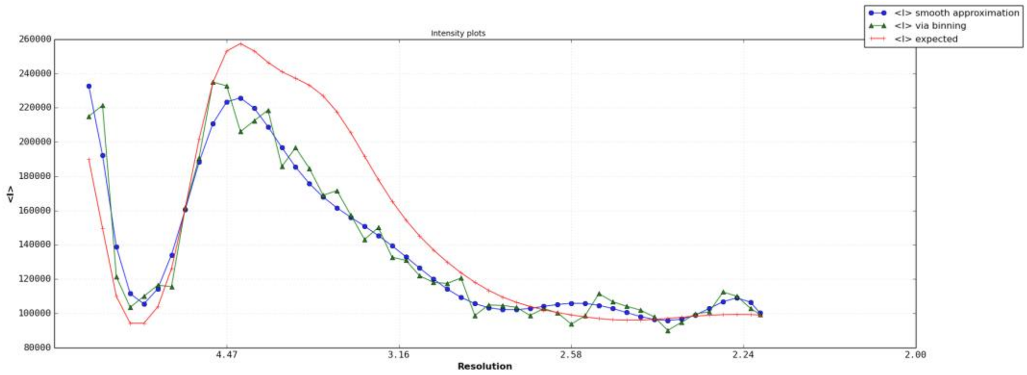

B

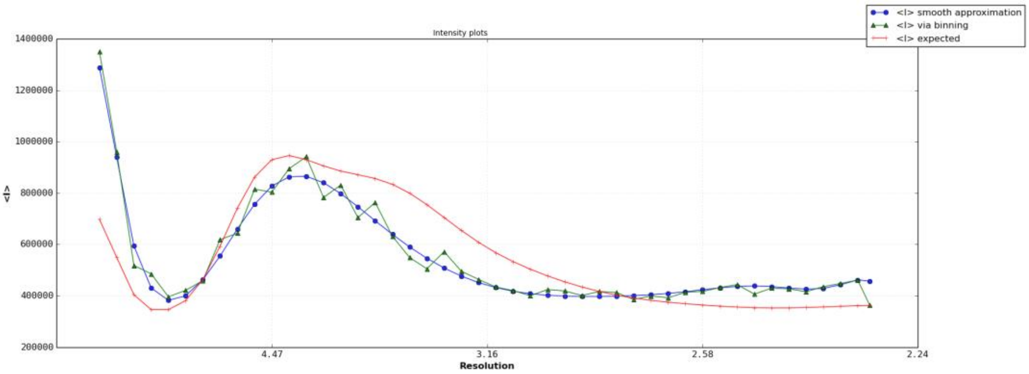

Supplementary Figure 8: Wilson plots (A) of the proteinase K data and (B) for the merged phycocyanin dataset measured with single pulse (Phyco_B). The graphs were prepared using phenix.Xtriage.

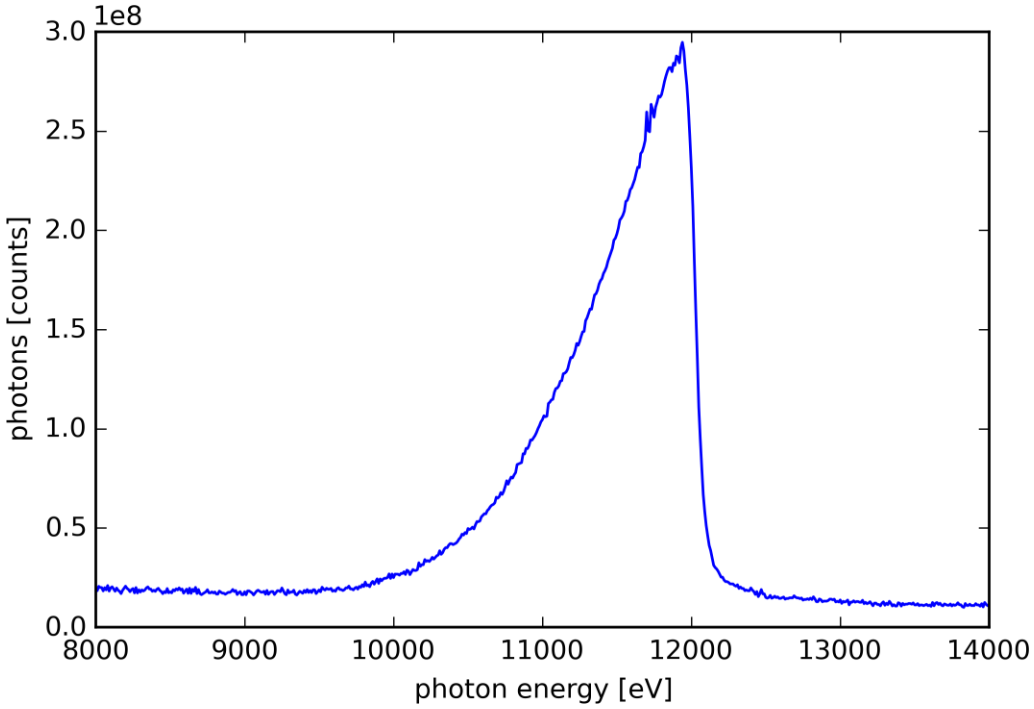

Supplementary Figure 9: Measured X-ray photon energy spectrum that was taken for the dose calculation. The total number of photons in the energy band of 9 keV to 13 keV is 3×10^{10} photons per pulse with a pulse energy of 60 µJ for the APS for the single pulse in the hybrid filling mode.

Supplementary Table 1: Comparison of our crystal structures derived from single pulse Laue data with structural data from single wavelength experiments in the protein data bank (PDB) reveals a lot of similarities and only some differences. Homologue protein structures are very similar to each other, as indicated by an overall rmsd value of below 0.2Å for main chain atoms of superimposed molecules. The biggest difference lays in the completeness of data sets, which is usually lower for Laue data due to challenging processing problems caused by spatial overlap of diffraction spots.

Proteinase K:
Only single crystal room temperature structures are available in the PDB, e.g. 2PRK at 1.5 Å and 4B5L at 1.6 Å resolution.

	5MJL (present work)	2PRK	4B5L
Resolution	2.21 Å	1.5 Å	1.6 Å
number of crystals	60	1 (0.7x0.7x0.9mm)	n.a.
photon bandwidth	5×10^{-2}	$\sim 10^{-4}$	n.a.
Completeness	61.9	n.a.	99.5
Redundancy	3.9	3.2	5.9
Number of reflections (ref) used in refinement	8342	30812	32791
Rwork	0.154	0.167	0.158
Rfree	0.196	n.a.	0.184
Rmsd angle	0.649	0.034	1.46
Rmsd bond	0.003	0.015	0.013
Mean isotropic B	6.2	11.1	14.9
Refinement software	Phenix	PROLSQ	Refmac

n.a. not available

Phycocyanin (Phyco_B, single pulse, multiple chips)

	5MJM (present work)	4ZIZ
Resolution	2.3Å	1.75
number of crystals	205	6679
photon bandwidth	5×10^{-2}	2×10^{-3}
Completeness	68.5	100
Redundancy	9.4	139.4
Number of reflections (ref) used in refinement	12119	40257

Rwork	0.139	0.204
Rfree	0.181	0.254
Rmsd angle	0.60	1.70
Rmsd bond	0.005	0.013
Mean isotropic B	22.9	n.a.
Refinement software	Phenix	Phenix

n.a. not available

Supplementary Table 2: Comparison of the statistics of the F_o-F_o-maps calculated from the respective phycocyanin datasets. Phyco_A1 and Phyco_A2 are all datasets merged from a single chip, collected in single (100 ps) pulse mode. For map calculation the resolution was limited to 2.70-15 Å, corresponding to the resolution range for Phyco_A2. Phyco_B is the dataset merged from 5 chips in single pulse mode, including Phyco_A1, Phyco_A2 and Phyco_C was merged from a single chip, collected in multibunch mode. For all maps the coordinates from 1JBO were used for phasing. Grey shaded fields indicate that these maps are shown in Supplementary Fig. 8.

F_O-F_O (CC_{iso}/R_{iso})	Phyco_A2	Phyco_B	Phyco_C	1JBO	4ZIZ
Phyco_A1	0.89/0.10	0.96/0.06	0.83/0.14	0.78/0.21	0.82/0.15
Phyco_A2		0.94/0.07	0.81/0.14	0.76/0.21	0.80/0.15
Phyco_B			0.84/0.13	0.79/0.22	0.74/0.15
Phyco_C				0.73/0.23	0.80/0.19
1JBO					0.80/0.25

Supplementary References:

1. Graber T, *et al.* BioCARS: a synchrotron resource for time-resolved X-ray science. *Journal of synchrotron radiation* **18**, 658-670 (2011).
